# Obituary

**Published:** 1905-08-15

**Authors:** 


### OBITUARY.

Dr. H. P. Teague, the managar of the Teague Dental Supply Co., of Augusta, Ga., died June 20th of consumption. Dr. Teague was a son of the editor of *Dental Hints*, and a graduate of the Atlanta Dental College. He practiced but one year and then organized the dental supply house which required all his time and attention up to the time of his death. He was a man of a happy and friendly disposition and was very popular where known. His death is greatly regretted by the members of the profession in the South, where he was widely known and loved.

